# A Few Remarks upon the Early Diagnosis of Pulmonary Tuberculosis*Read before the Buncombe, N. C., County Medical Society, October 4, 1897.

**Published:** 1898-04

**Authors:** C. P. Ambler

**Affiliations:** Asheville, N. C.


### A FEW REMARKS UPON THE EARLY DIAGNOSIS OF PULMONARY TUBERCULOSIS.*

By C. P. AMBLER, M. D., ASHEVILLE, N. C.

We speak and read of the early stage of pulmonary tuberculosis as though it were some well-defined and easily recognized condition; whereas, in fact, the term is used by different observers, in describing the clinical history of a case, with the greatest latitude and often with little knowledge or respect for the true condition of the patient.

As to what constitutes an "early-stage case" there seems to be the widest difference of opinion. It is the difficulty that various observers experience in following a standard as to classification of patients that makes the statistics of cases published as cured so unreliable. One observer may use the greatest care in classifying, with the result that he shows a large percentage of cures in a given class. Another observer does not discriminate so carefully, fails to get as good a percentage cured, doubts the veracity of the first observer, creates discord and incredulity among the profession, and thus not only damages the reputation of the profession, but creates distrust in the minds of the public generally.

Before Koch discovered the tubercle bacillus a case was almost always classed as an early-stage case, provided the general appearance of the patient remained fair. Since that time we have learned to rely more upon certain physical signs, we have been much more thorough in our examination, and while we still rely upon the microscope to confirm our diagnosis absolutely, we are beginning to recognize that there is a possibility of making a diagnosis before the stage of expectoration has been reached.

Before Koch made this discovery but comparatively few cases were recognized as tuberculosis before they were hopelessly advanced; the physician having at hand no absolute means of diagnosis, he was perhaps prone to give the patient the benefit of the doubt, and with the results following it is no wonder that the laity to this day consider the disease incurable.

The profession to-day have, almost without an exception, recognized that the disease is curable, the prognosis depending in great part upon the diagnosis being made early; it will take the laity generations to realize and appreciate this fact.

I believe the profession of Asheville will bear me out in the statement that but comparatively few of the many cases sent here are what we should call early-stage cases.

It has been my experience that the early-stage cases met with here are in the persons of friends or relatives accompanying some more advanced patient. Becoming fearful that

*Read before the Buncombe, N. C., County Medical Society, October 4, 1897.

their association with tuberculous patients may have resulted in infection to themselves, and after being instructed upon the advantages and necessity of an early diagnosis, they present themselves for an examination, with the result that we frequently find evidence of a morbid process going on in the lungs unsuspected.

So-called first-stage cases usually embrace all cases up to the point of moderate infiltration in the lung, and possibly bordering on active cavity formation, without the advanced general symptoms.

As our experience in this disease grows we are becoming dissatisfied with the old classification. The first or early stage is being limited nearer to the time of infection; our early-stage cases to-day are those recognized before the stage of expectoration.

We have long recognized such a condition, and the tendency has been to call this a pretubercular state, or to convey to the patient the impression that he is threatened with tuberculosis.

Statistics from all observers go to show that those cases recognized during this period, or immediately following it, almost invariably do well, regardless of what certain line of treatment is followed, provided certain hygienic laws are observed.

Unfortunately, a large percentage will not consult us until the disease is well advanced; here we can, at least by the exercise of a little patience, early determine the true state of affairs. It would seem that no physician is excusable where a proper diagnosis is not made when the period of expectoration has been reached.

That such mistakes are made, however, we all know—made, too, by carelessness on the part of the physician, following a hasty physical examination; made often without removal of the patient's clothing. Without the use of a stethoscope, without the use of a fever thermometer, without a microscopical examination of expectorated matter, an opinion is given that the patient has "bronchitis," a "weak lung," or an ordinary "cold." The opinion coming from a physician in whom the patient has certainly some confidence, and often considers a smart man from his being able to so quickly pronounce his lungs sound, he goes off, takes the tonic prescribed, and for weeks goes about his business, losing his golden opportunity, relying upon the opinion given that he "will shortly be all right."

Again, he consults some timid brother of ours who, by the exercise of more care in his examination, arrives at the proper conclusion, and who, through mistaken kindness, does not tell him the truth; advises him to go to Asheville, that his lungs are weak—leaving for the Asheville physician to tell him his true condition, when, if he had been told the truth at once, he would have exercised more care and had more chance for recovery.

It is not the purpose, however, of this short paper to show why mistakes are made in diagnosis through carelessness on the part of the physician, but more as a plea for earlier diagnosis and to bring out a discussion as to the possibility of making a diagnosis before the stage of expectoration, at which time any careful observer is bound to arrive at the proper conclusion.

I have for years advocated that when a physician is attending a case of pulmonary tuberculosis he should, at least once a year insist upon a physical examination of each member of the family, regardless of the subjective symptoms. Supposing, of course, the physician to be proficient in making a physical examination, he is certain, sooner or later, to find suspicious symptoms of a morbid process in the lungs of certain individuals. When his suspicions are aroused let him follow up the case carefully, and such cases as are tuberculous will have a much better prognosis for their being thus early determined.

I am informed by a cousin of mine, who has lived in Japan for years, that in certain parts of the empire the physician is free to examine the premises or any member of the household where he is employed at any time; he is paid a certain fee every year, provided no sickness occurs, and in case it does occur, he is compelled to attend the case without charge for the period during which the sickness continues, the disease being supposed to be the result of his neglect.

The subjective symptoms present before the stage of expectoration are so indefinite, if any exist, but that little importance can be attached to them. An exception to this occurs, however, when the patient has a hemorrhage from his lungs as the first indication of lung trouble, or where a dry, chronic cough persists without apparent cause being found in the throat.

Pain, unfortunately, is rarely, if ever, complained of at this time. Unfortunately, I say, because if it were the rule, instead of the reverse, we would see our patients much earlier.

There can be no doubt that certain objective symptoms are always present long before the patient begins to expectorate, and if the physician had the opportunity of examining the case at this time he might demonstrate the same; moreover, if the physician had some means of coming in contact with the patients earlier it is quite likely that such clinical history would have resulted long ago in a better appreciation of the symptoms present at this the earliest stage of the disease. If, after a rigid examination, we are able to detect a slight relative dullness upon percussion; if we can determine a circumscribed area of harsh, rough, or bronchial breathing; if we find prolonged expectoration or interrupted respiration; if we can detect a suspicion of rales; if we can demonstrate either of them alone or associated one with the other, we should strongly suspect some morbid process, and, watching our patient carefully, should never relax our efforts until we have proved to our entire satisfaction that no tuberculosis is present.

Having our suspicions aroused, how shall we proceed to demonstrate that our supposition is correct; or, rather, how shall we proceed to show that no tuberculous deposit is present? First, and always, let us be on our guard against making a hasty diagnosis, or expressing an opinion before the patient which will in any way be construed by him as meaning that the trouble is slight. If you so assure him now, you will very likely have no further opportunity for investigation until he returns to you well advanced in the tubercular process.

In the absence of stronger evidence than above enumerated, Koch would have us give our patient a test dose of tuberculin. Experience has shown that it will assist materially in clearing up a doubtful diagnosis under such circumstances; but will not a drug which is capable of exciting a sudden rise of temperature from one to four degrees above the normal, when placed in the circulation and thus brought directly in contact with a tuberculous deposit, at the same time excite the deposit, which may be latent, into a more active process? Or, may it not, and, in fact, does it not, only produce this rise of temperature by the local congestion or inflammation of the diseased area? Are you willing to risk the consequences? Do you believe you would carry out such a procedure in your own person under such possibilities? If you would not, you have no right to use it upon your patients.

Such a method may do for testing cattle, but it will be slow in becoming a popular method with our profession. It is like hunting for a gas leak with a lighted candle.

Attempts are now being made to show that a blood examination at this early period will reveal an existing tuberculosis as certainly as will the presence of bacilli in the expectoration later on. Such a method would have everything in its favor. We trust there may be much in it, but in our ignorance we doubt it.

Being unwilling or unable to use either of these methods, we still have great resource in the temperature of the body. I have never seen a statement as to the time the temperature begins to deviate from the normal in pulmonary tuberculosis. This much I know, however, from my own observation, that it is not unusual for a slight daily rise to occur before the stage of expectoration has been reached.

To be of any service whatever the temperature record must be taken with the greatest care; the temperature should be observed every two hours for a period of several days, and an exact record of the same kept for comparison. A continuous normal temperature for several days or weeks does not exclude tuberculous deposit, but a slight rise of one or one and a half degree daily, when existing in connection with certain physical signs as enumerated above, does go far toward confirming our suspicions. Suppose such a rise of temperature is found associated with nothing more than slight relative dullness or harsh breathing over a circumscribed area in the lung; the symptoms continue for weeks, with perhaps intervening days when the temperature is normal; no other cause can be found. You are forced to reason by exclusion. What do you conclude? What can you do but conclude that you have a tubercular deposit at its early stage?

Dr. Babcock, of Chicago, at the last meeting of the American Climatological Association, held in Washington this summer, stated, in discussing this subject, that "a variation of one half-degree or one degree from the normal daily temperature caused but little import." My experience certainly does not confirm this.

In all cases where I have observed the patient for weeks preceding the stage of expectoration, I have invariably found a fluctuation of the daily temperature.

At a recent meeting of the society I alluded to the fact that we occasionally meet cases where the question of diagnosis between early tuberculosis and a beginning or mild type of typhoid fever was difficult to determine until after the lapse of several days. At that meeting one of the members took exception to what I said, and remarked that the symptoms present in typhoid fever were always sufficient to enable one to make a diagnosis I wish to repeat that I do not believe all cases of typhoid fever must necessarily be severe in form, and, further, that the symptoms of a mild case, especially in the beginning, are in some respects similar to those observed in early tuberculosis. Moreover, the acute exacerbations of a tuberculous fever in the so-called first stage are sometimes mistaken for symptoms of other diseases, notably a common cold or typhoid fever, and are treated as such.

Let us remember that an early diagnosis is of more value, in reference to prognosis, than the administration of drugs.

So far as possible, let us examine the members of families we treat, especially so when some member is already suffering from tuberculosis, and, finally, let us remember that certain physical signs, when found concomitant with a daily rise of temperature over a series of days or weeks, are strongly indicative of tuberculosis, and, where the slight rise of temperature can not be otherwise satisfactorily accounted for, no hesitation should be entertained in pronouncing the condition probably one of tuberculosis.—*New York Medical Journal.*

